# The lithological characteristics of natural gas hydrates in permafrost on the Qinghai of China

**DOI:** 10.1038/s41598-022-17475-7

**Published:** 2022-08-02

**Authors:** Da Lei, Changmin Fu, Qihui Zhen, Zhongxing Wang, Ruo Wang

**Affiliations:** 1 CAS Engineering Laboratory for Deep Resources Equipment and Technology, Institute of Geology and Geophysics, Chinese Academy of Sciences, Beijing, 100029 China; 2 Institute of Geology and Geophysics, Chinese Academy of Sciences, Beijing, 100029 China; 3 College of Earth and Planetary Sciences, University of Chinese Academy of Sciences, Beijing, 100049 China; 4 Innovation Academy for Earth Science, Chinese Academy of Sciences, Beijing, 100029 China

**Keywords:** Geophysics, Solid Earth sciences

## Abstract

The environment is seriously threatened by the methane emitted as permafrost melts. Studying deposits of natural gas hydrates that include methane is therefore important. This study presents a novel approach based on the rock Archie formula to discover the porosity and saturation of gas hydrates. The relationship between resistivity and porosity and the porosity of hydrates was studied, and the results showed that the resistivity of hydrate reservoirs was closely related to porosity and hydrate saturation, and the polarization rate was only related to the concentration of natural gas hydrates and had nothing to do with porosity. Using the multi-channel time domain induced polarization (MTIP) method, the profile with five boreholes in the Muli area of the permafrost area of the Qinghai-Tibet Plateau was observed, and the thickness of the shallow permafrost distribution and the underground structure were inferred based on the resistivity of the MTIP data. The polarization rate and hydrate saturation of the inversion assessed the presence of hydrates in the Muli region. The results show that the MTIP method can be used to detect the thickness of permafrost distribution, determine fault boundaries, reveal the distribution of natural gas transport paths, and evaluate the presence of natural gas hydrates.

## Introduction

Gas hydrates are crystalline minerals that are made up of water and various gases. They are discovered in large quantities, mainly on the seafloor and in regions with permafrost^1,2^. Hydrates are a source of combustible energy. Estimated global reserves are 2.1 × 10^15^ m^3^, twice the total reserves of coal, oil, and natural gas combined^3,4^. Therefore, countries all over the world, especially developed countries and those with energy shortages, have attached great importance to research on natural gas hydrates (NGH). The United States, Japan, Germany, India, and Canada have established institutes to research NGH as well as development plans to expedite the exploration, development, and utilization of their resources. Based on research in these countries, nine permafrost regions containing NGH have been identified. They are located in Russia, the United States, Canada, and countries in the permafrost zone of the Central Arctic^5^. China has the third largest permafrost deposits of NGH in the world, which are on the Qinghai-Tibetan Plateau and the Greater Xingan Mountains, accounting for approximately 22.3% of China’s reserves^6^. The permafrost region of Qilian Mountain, China, is located on the northern edge of the Qinghai-Tibet Plateau and is primarily composed of mountain permafrost. The average annual surface temperature of the continuous permafrost area is − 2 to − 2.5 °C, and the frozen soil layer thickness is 60–95 m^6^. It can provide advantageous temperature and pressure conditions as well as an excellent trap effect for the formation of NGH.

In marine surveys, seismic techniques have been used to locate NGH in sediment under the stable zone of the seabed^7–24^. Seismic methods are used to predict the amount of methane in the saturated hydrate in the pore space of NGH reservoirs^25–31^. The controlled-source electromagnetic method has been used to determine the pore saturation^30–32^. The seismic reflection method has proven to be effective against NGH exploration in permafrost^33,34^, whereas electromagnetic methods have only recently been used in the field to study hydrate deposits^35,36^.

In recent decades, the China Geological Survey Bureau has supported research on NGH in permafrost regions. In 2008 and 2009, research was carried out in the permafrost region of the Qilian Mountains along the northern edge of the Qinghai-Tibetan Plateau, which has conditions suitable for NGH. An NGH Scientific Drilling Project was carried out in the Qilian Mountains. Boreholes DK-1, DK-2, DK-3, DK-4, and DK12-13 were drilled, and sufficient rock samples of gas hydrates were obtained to give rise to scientific and economic importance^37,38^. An electromagnetic method^39^ has been used for NGH exploration in China since 2009. We have further evaluated hydrates in a typical section in the Qilian Mountains Muli region using multichannel time-domain induced polarization (MTIP) to determine the distribution of permafrost, source rocks, and transport channels of hydrates, as well as the distribution of hydrates delineated according to polarizability and NGH saturation, providing methodological support for an in-depth understanding of the distribution pattern and resource potential of gas hydrates in the area.

## Study area

### Geological background

**Figure 1:**
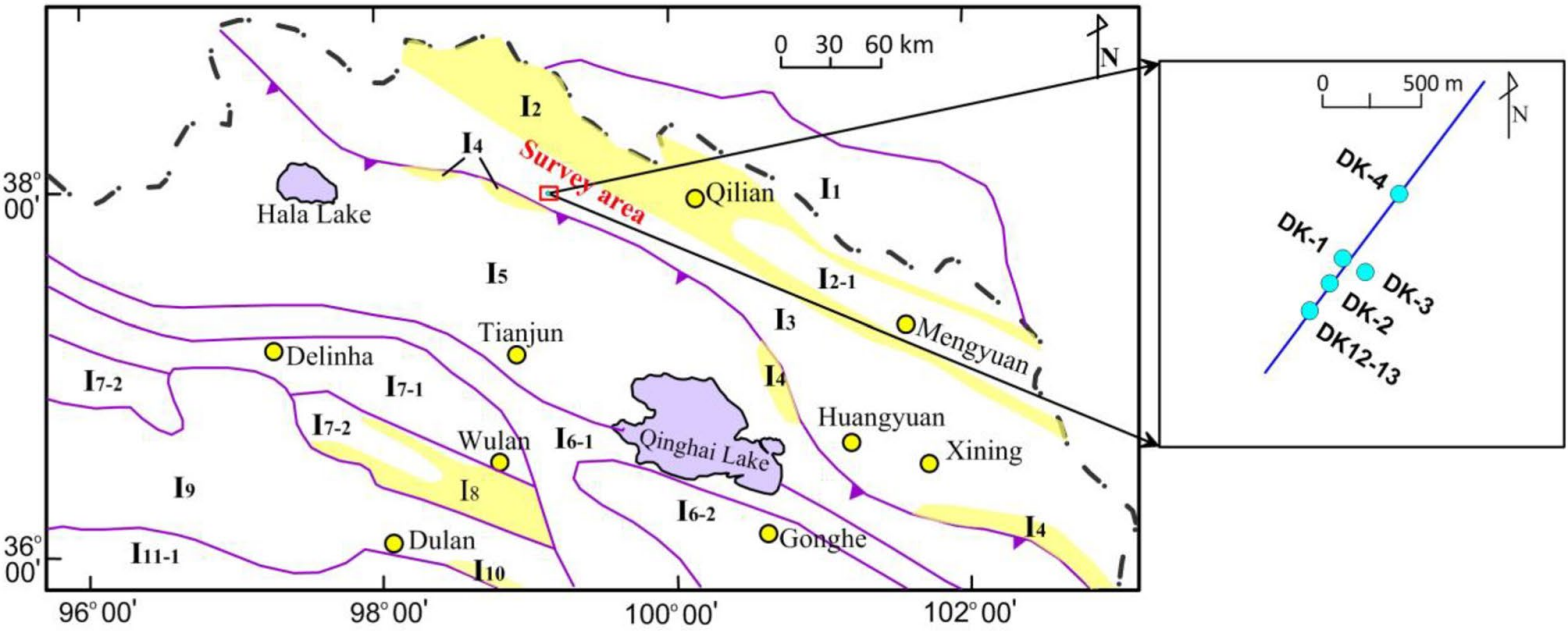
Schematic diagram of structures within the study area overlaid on an aerial photograph. I_1_ represents the Alxa landmass; I_2_ represents the Northern Qilian neo-Proterozoic to early Paleozoic suture zone. I_2-1_ represents the Qilian-Menyuan magmatic arc zone, middle and late early Paleozoic (O-S). I_3_ represents the Qilian block in the center. I_4_ represents the Suture zone between Shule Nanshan and Laguiyama in the Early Paleozoic. I_5_ represents the Southern Qilian's landmass. I_6_ represents the Late Paleozoic-Early Mesozoic Fracture Trough (D-T2) at ZongwuLongshan-Qinghai Nanshan. I_6-1_ represents the Zongwu Longshan-Xinghai Aola Trough (D-P). I_6-2_ represents the Post-Foreland Basin of the Zeku Arc (T_1-2_). I_7_ represents the Oulongbrook Land Block. I_7-1_ represents the Orbo Mountain, Craton Edge Basin. I_7-2_: T-shaped VI-Amunik Mountain-Depleted Late Neoproterozoic Niushan Magma Arc Belt (Pt_3_-S). I_8_ represents the ChaiBei Rim's Suture Zone. I_9_ represents the Qaidam's Plot. I_10_ represents the Qimantag-Dulan Suture Zone. I_11_ represents the Dongkun Middle Land Block. I_11-1_ represents the Dongkun Middle Magma Arc Zone (Pt_3_-J). The blue dots in the box on the right represent the drilling wells within the survey area (Modified from Lu et al.^7^, 2010. Map created in Surfer 14.0.599 of Golden Software, LLC (https://www.goldensoftware.com/products/surfer).

According to^40^, the Qilian Mountains are in the northeast of the Qinghai–Tibet Plateau, China. There are three major tectonic units: the north Qilian tectonic belt (Hexi corridor and South Mountain corridor), the middle Qilian continental block (Tolai Mountain), and the south Qilian tectonic belt, which correspond to I2, I3, and I5 in Fig. 1, respectively. The main body of the southern Qilian tectonic belt is a superposed basin from the late Paleozoic to the Mesozoic, which developed by early Paleozoic tectonic evolution.

Boreholes DK-1, DK-2, and DK-3 were drilled in the town of Tianjun in Qinghai Province, in the permafrost regions of Muli, which are at an elevation of between 4026 and 4128 m. The three holes revealed a permafrost thickness of approximately 95 m^41^ and an average annual surface temperature of approximately − 2 to − 2.5 °C in the area, with the main drilling area being in the South Qilian structural belt, which is subordinate to the Muli Depression^42,43^.

The central part of the study area is composed of anticlinal Triassic strata, and in the north and south, there are two synclinal Jurassic coal-bearing strata. The large-scale thrust nodes on the north and south of the anticline control the boundary of the depression. The north–south synclines have caused a series of large shear faults in the northeast that cut the depression into intermittent segments of different sizes (Fig. 1). The boreholes reveal that strata within the study region contain the Jurassic Jiangcang Formation (J_2_j) and Muli Formation (J_2_m), but not the Quaternary system. The Muli Formation roughly corresponds to the Xiangtang Formation (J_2_x) and the Yaojie Formation (J_2_y) in this region.

Lu et al*.*^40^ claim that there are several recoverable coal seams in the strata mentioned above. The Jiangcang Formation (J_2j_) is dominated by black and gray oil shale, mudstone, gray sandstone, and fine sandstone. The Muli Formation (J_2_m) is dominated by gray and gray-white siltstone, fine sandstone, medium sandstone, coarse sandstone (gravel), deep gray mudstone, and oil shale, which are sediment from a braided river delta and the main coal-bearing section. It contains two major coal seams and several local thin coal seams. However, the hydrate is mainly distributed in the mudstone, siltstone, oil shale, and fine sandstone. It is between 130 and 400 m deep in rock fractures that may not be visible to the naked eye. It appears as an abnormality in finely disseminated deposits distributed in rock pores. These strata belong to the Jiangcang Formation.

### Electrical and lithological characteristics of NGH

The MTIP survey carried out in the permafrost region of the Qilian Mountains was based on differences in resistivity between the targeted geological bodies (e.g., permafrost and structural faults) and the surrounding rocks. Gas hydrates occur in fissures of siltstone, mudstone, oil shale, or in pores of sandstone. The content of organic carbon in the oil shale is 0.98–5.76%, which satisfies the standard for high-quality source rock^44^. Oil shale has entered its mature period and is the main source of gas^40,45^.

NGH is unstable under normal temperatures and pressure, and thus, it is difficult to determine its physical characteristics by collecting samples. However, it is not difficult to analyze the characteristics of the resistivity of NGH and permafrost using in-situ measurements from well logging. An analysis of log data from this area revealed that the NGH and permafrost have a higher resistivity than the normal sedimentary strata.

**Figure 2:**
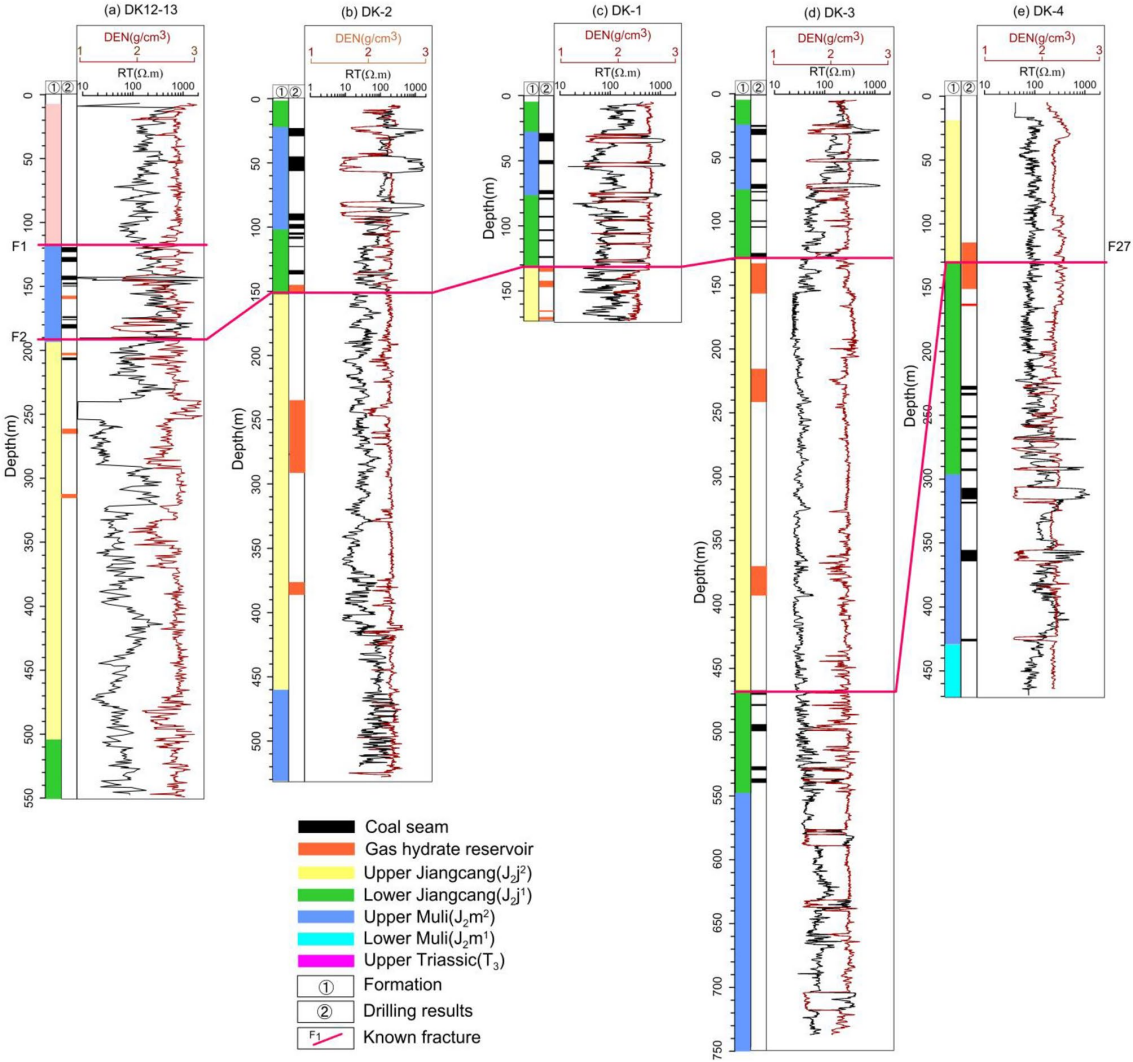
Resistivity versus depth from well logs: (**a**) DK12-13, (**b**) DK-2, (**c**) DK-1, (**d**) DK-3, and (**e**) DK-4.

Figure 2a–e, respectively, show the logs of borehole resistivity from wells DK12–13, DK–2, DK–1, DK–3, and DK–4 in the Qilian Mountains. The figures demonstrate that the resistivity of layers of sandstone, shale, siltstone, oil shale, and mudstone, within which the NGH was mainly deposited, ranged from 133 to 283.7 m, and from 314 to 396 m, respectively.

The gas hydrate-bearing layers show obvious high resistivity anomalies in the resistivity logs of DK-1 and DK12–13, while other log resistivity curves have weaker displays. According to the lithological characteristics of five well logs, the resistivity values of hydrate-bearing layers are statistically classified in Table 1. It can be seen in Table 1 that NGH revealed by well DK-1 exists in sandstone and siltstone. The mean resistivity value of the hydrate gas-bearing layers is 3.35 times higher than that of the surrounding rock. The NGH revealed by well DK12-13 exists in siltstone, shale, and mudstone, and the mean resistivity value of the gas hydrate-bearing layers is 2.30 times higher than that of the surrounding rock. The NGH revealed by wells DK-2, DK-3, and DK-4 exists in mudstone, siltstone, and oil shale, and the mean resistivity value of the gas hydrate-bearing layers is 1.70 times higher than that of the surrounding rock. The mean resistivity value of the NGH layers in five holes is 2.26 times higher than that of the surrounding rock. It is consistent with the conclusion pointed out by Fang et al.^39^ that the resistivity of the gas hydrate layer is two to three times higher than that of the surrounding rock.

**Table 1:** Statistics of resistivity values of NGH Reservoirs from resistivity logging.

Borehole	Reservoir lithology	Gas hydrate bearing layers (m)	Resistivity value of surrounding rock (Ω m)	Reservoir resistivity value (Ω m)
DK-1	133.5–135.5	Sandstone	53.85–61.90	203.73–378.41
	142.7–147.7	Siltstone	52.85–88.05	146.41–349.92
	165.45–166.55	Siltstone	30.44–34.21	49.48–96.99
	169.0–170.5	Siltstone	55.98–76.96	101.94–242.12
DK-2	144.40–156.6	Shaly sand	19.42–64.39	28.39–182.73
	275.8–277.1	Siltstone	29.55–36.69	45.75–70.08
	282.5–283.7	Mudstone, siltstone and oil shale	46.08–56.70	78.22–98.93
DK-3	133.0–156.0	Mudstone, siltstone and oil shale	25.67–30.72	40.29–86.16
	225.1–240.0	Mudstone	29.86–35.5	25.37–47.95
	367.7–396.0	Mudstone	24.87–27.91	24.17–59.64
DK-4	115.0–150.0	Siltstone, mudstone	70.16–77.54	57.87–153.70
	162.0–163.0	Siltstone, mudstone	59.71–61.51	66.44–84.22
DK12-13	157.5–160.3	Siltstone	91.11–210.21	260.3–396.6
	201.3–203.5	Shaly sand	37.3–56.4	80.19–170.12
	263.2–265.4	Mudstone	12.17–20.21	30.03–55.89
	314.0–316.1	Siltstone	120.02–183.67	200.89–300.51

Comprehensive information from the drilling and cores shows that the NGH mainly occurs in the Jiangcang formation in the Middle Jurassic of the Muli permafrost.

In well DK-1, porosity measurements from core samples of the four wells ranged from 5 to 20%. The range of NGH saturation obtained by the Archie equation is 13–86%^46,47^. In wells DK-2 and DK-3, the mean value of NGH saturation obtained by the Archie equation is 9.5% and 15.5%, respectively^48^. Well DK12-13, the range of NGH saturation achieved by the Archie equation is 13–85%^49^. Therefore, the porosity of the rocks in the four wells varied from 5 to 20%, and the saturation varied from 13 to 86%.

The reservoir resistivity range of the gas hydrate-bearing layers is the minimum and maximum values of the corresponding logging resistivity curves, and the surrounding rock resistivity range is the minimum and maximum values of the logging resistivity curves corresponding to the upper and lower formations of NHG-bearing reservoirs.

### MTIP sounding layout

**Figure 3:**
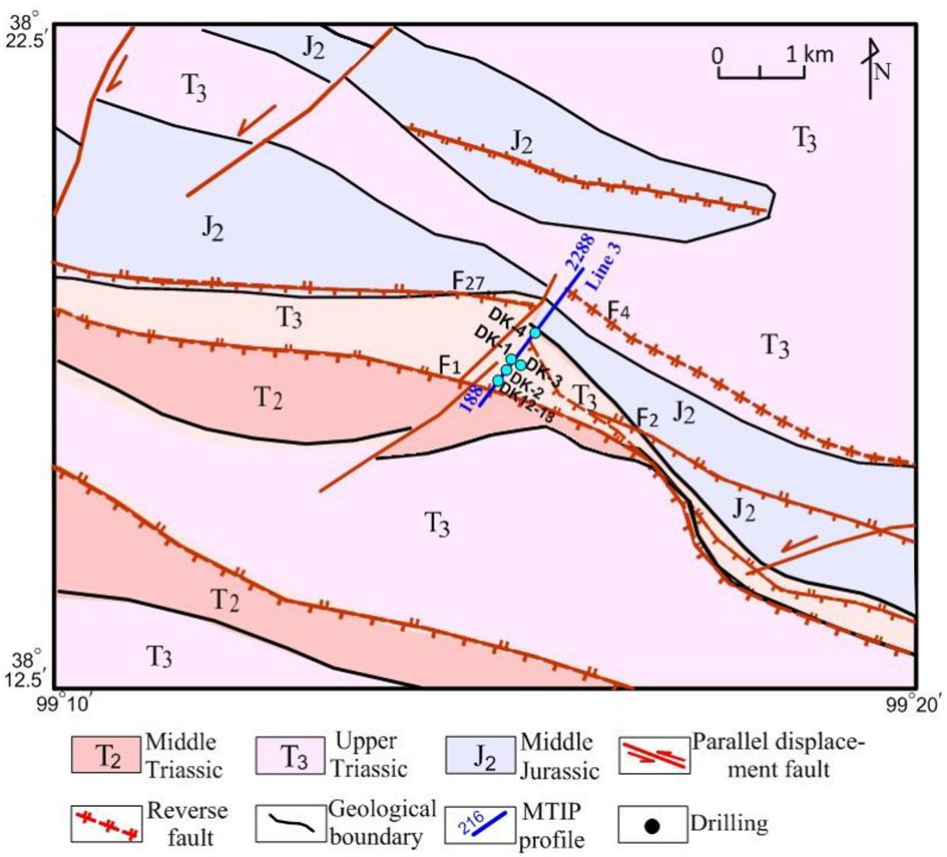
Geological map of the research area; positions of MTIP survey lines; with MTIP sounding profile (blue). Map created in Surfer 14.0.599 of Golden Software, LLC (https://www.goldensoftware.com/products/surfer).

An experimental study of the MTIP sounding method for the detection of NGH has been ongoing in the Muli area since 2008. The survey lines are shown in Fig. 3. Line 3 was across wells DK-4, DK-3, and other gas hydrate investigation wells, which were 2100 m long. In the pole–dipole setup, the dipole spacing was used at 20 m.

## Methods

### The MTIP principle

**Figure 4:**
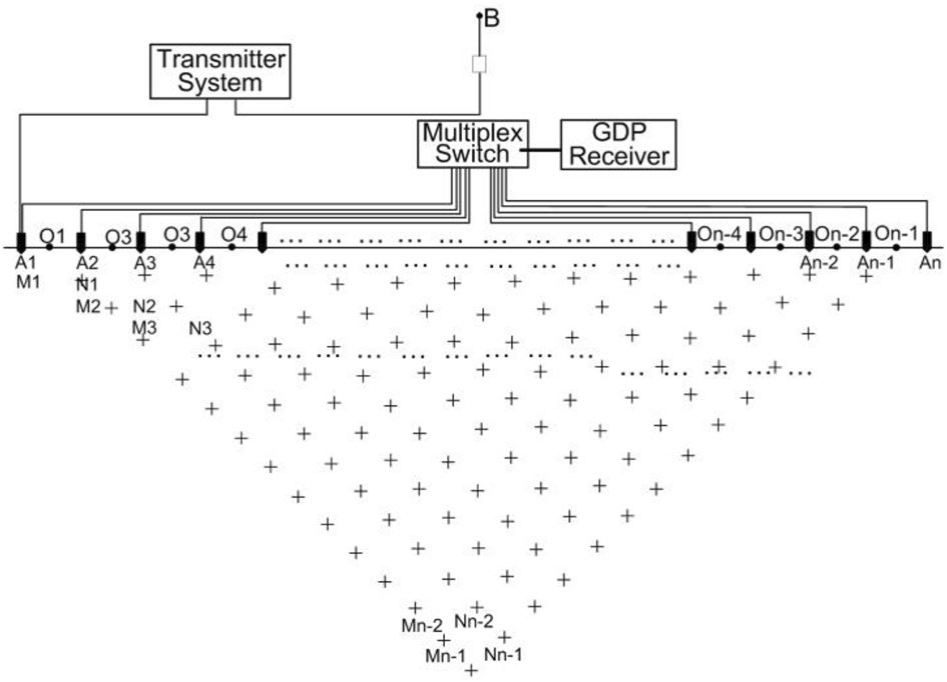
Setup of the MTIP survey.

MTIP is an array exploration method based on the difference between in conductivity and polarizability between the study object and the surrounding rock and the distribution of the conduction current underground under the action of an artificially stabilizing current field^50^. The survey diagram is shown in Fig. 4. It is a time-domain-induced polarization method. As with conventional ECR with polarization, all receiving electrodes and receiving wires on a profile are laid out prior to measurement, and pole-dipole devices are used for observation. However, the difference is that our team's multi-purpose GDP electrical system (Zonge Ltd., USA) It was developed to be used with an 8-channel transfer switch developed to observe the data through the transfer switch. This allows the use of GDP's high-power transmitter and high-precision data acquisition device for deep apparent resistivity and polarization measurements. The distance between the measuring points and the electric dipole moment can be flexibly varied depending on the depth. Therefore, MTIP resistivity and polarizability imaging is a detection method with large depths (10–800 m).

### MTIP data processing

The 2D inversion software TS2DIP 4.40b (Zonge Engineering & Research Organization, Inc., USA. https://zonge.com.au/what-we-do/data-processing) was used for MTIP data inversion. A smoothing model inversion is a robust way to convert resistivity and polarizability data into a smoothly varying model profile. The finite element forward-modeling algorithm used in TS2DIP calculates the apparent resistivity and polarizability with an accuracy of 5% from a 2D model. When information about the terrain is included in the model, the terrain is clearly reflected in the finite element mesh of TS2DIP. Average values of the apparent resistivity and polarizability were calculated and used in the initial background resistivity model. The interactive tool allows the user to edit the background model autonomously based on known geological information. The iterative modification of the 2D model was guided by constraints on both its smoothness and the differences between the background model and the inversion model. This method considered many measures, including the RMS error, to measure data misfit, distance from an a priori background model, model roughness, average RMS model-constraint residual, RMS minimization criteria, and the largest changes in the model parameters after each iteration until the calculated resistivity and polarizability matched the observed data as closely as possible.

### Porosity and saturation calculation methodology

In order to use MTIP to explore the NGH in the Muli area of the Qinhai-Tibetan Plateau, it was necessary to study the lithological characteristics based on resistivity and polarizability.

The physical parameters affecting the electrical properties of rocks in the area containing NGH are the porosity and saturation of the gas hydrate. Archie’s equation^51^ is commonly used to evaluate a reservoir and can be applied to NGH:

1 $$\rho_{t} = \frac{{a\rho_{w} }}{{\phi^{m} S_{w}^{n} }},$$

where *ρ*_*t*_ is the resistivity of the formation (Ω m), *ρ*_*w*_ is the resistivity of the water in the formation (Ω m), and *ϕ* is the porosity (percentage). It is generally believed that the pores of hydrate-bearing reservoirs contain only hydrates and water, *S*_*w*_ is the saturation of pores in the formation due to water and gas hydrate saturation *S*_*h*_ is obtained by:

2 $$S_{h} = 1 - S_{w}$$

The parameters *a*, *m*, and *n* are empirical indices that can be determined for the stratum. In general, 1.5 < *m* < 3, 0.5 < *a* < 2.5, and 1.86 < *n* < 2.06^46^.

According to past research on NGH reservoirs^46,52^, *ρ*_*w*_ = 2 Ω m, *n* = 1.9386, *a* = 0.51, and *m* = 1.32, so Eq. (1) can be written as

3 $$\rho_{t} = \frac{1.02}{{\varphi^{1.32} (1 - S_{h} )^{1.9386} }}$$

Equation (3) shows that the resistivity of a NGH reservoir is a function of the porosity and saturation of the NGH. Thus, the resistivity of the NGH reservoir can be deduced from these two parameters in the study area.

In the time-domain IP method, the measured voltage in the rock and ore increased over time with a stable current, indicating that the resistivity of the rock and ore or NGH changed with supply time. In other words, the effect of volumetric polarization of the medium is equivalent to the increase in its resistivity when the supplied current is stable. The equivalent resistivity of the IP is given by Seigel^53^.

4 $$\eta = (\rho_{t} - \rho_{0} )/\rho_{t} = 1 - {{\rho_{0} } \mathord{\left/ {\vphantom {{\rho_{0} } {\rho_{t} }}} \right. \kern-\nulldelimiterspace} {\rho_{t} }} = 1 - \frac{{\varphi^{1.32} (1 - S_{h} )^{1.9386} \rho_{0} }}{1.02}$$

where *ρ*_*t*_ is the resistivity of the formation (Ω m), *ρ*_*0*_ is the resistivity of non-excited electricity generation when the water content is zero, and *η* is the polarizability (percentage). Therefore, the polarizability can be estimated with Eqs. (3) and (4). The resistivity calculated according to porosity and NGH content is the equivalent resistivity, and the resistivity calculated without the NGH is the resistivity without excitation. Knowing the resistivity and polarization rate, *ϕ* and *S*_*h*_ can be obtained by solving together with Eqs. (3) and (4).

## Results

### MTIP sounding results

**Figure 5:**
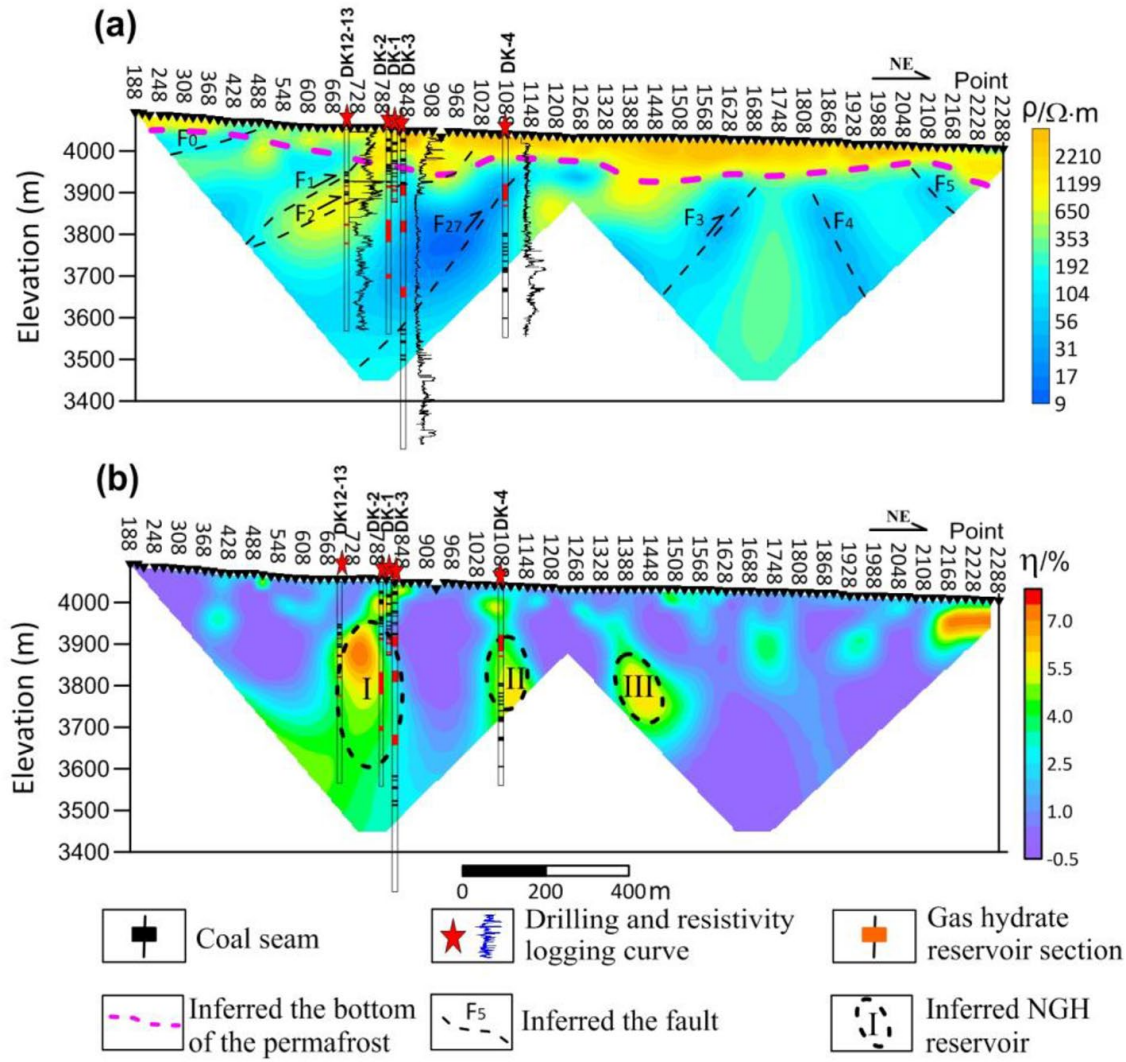
**I**nferences from well logs and inversion sections of MTIP data. (**a**) Section of two-dimensional inversion resistivity; (**b**) Section of two-dimensional inversion polarizability.

Figure 5a shows the two-dimensional resistivity inversion section of MTIP data. It reflects the details of these resistivity logs, especially the high-resistivity anomaly (650 Ω m or more) between depths of 0 and 150 m for the section, which is consistent with the resistivity logs of DK-3 and DK-4. The high-resistivity anomaly shows that there was a layer of frozen soil within the shallow part of this section, and the thickness of the point measurement reaction near the boreholes DK-1, DK-2, and DK-3 coincides with the thickness of the known permafrost layer of about 95 m^41^. The resistivity logs of DK12–13, DK-3, and DK-4 indicate the presence of a lower resistivity region between depths of 200 and 590 m, 100 and 600 m, and from 70 to 260 m, respectively. The low resistivity region was also observed in the resistivity section. NGH reservoirs are distributed in this region. NGH in the Muli area mainly occurs in fractures of mudstone or oil shale, which causes the inclined low resistivity zone of inclined mudstone and the middle-high resistivity anomaly of the NGH reservoir. The results show that the section has seven faults: (a) five south-dipping faults (F0, F1, F2, F27, and F3) and two north-dipping faults (F4, and F5), which reflect the low resistivity seen in MTIP data. (b) MTIP data revealed two north-dipping faults (F4 and F5) associated with low resistivity. The results indicate that the F1, F2 and F27 fracture zones control the formation of NGH. This is consistent with geological and drilling findings that F1, F2 and F27 faults are migration channels for NGH and accumulation spaces for NGH. However, it is difficult to distinguish the NGH layers in the two-dimensional MTIP resistivity inversion section. There are two main reasons for these blind spots. First, the NGH layer is small and it is difficult to identify the deposit with the available detection precision. Secondly, the NGH layer is close to the permafrost layer or close to the faults; hence, the difference in resistivity within the region is very small.

Figure 5b shows the two-dimensional polarizability inversion section for MTIP. There are many high-polarizability anomalies in the section. I, II, and III are inferred ranges of NGH reservoirs. There is a correlation between the known ore-bearing sites and the high-polarizability anomaly I between depths of 190 m and 425 m for DK12–13; depths of 145 m and 390 m for DK-2 and DK-3, respectively; and the high-polarizability II between depths of 145 m and 395 m for DK-4. The I and II high polarizability anomalies are located near the F1, F2 and F27 faults. The high-polarizability anomaly III is near the F3 fault.

### Porosity and NGH saturation

**Figure 6:**
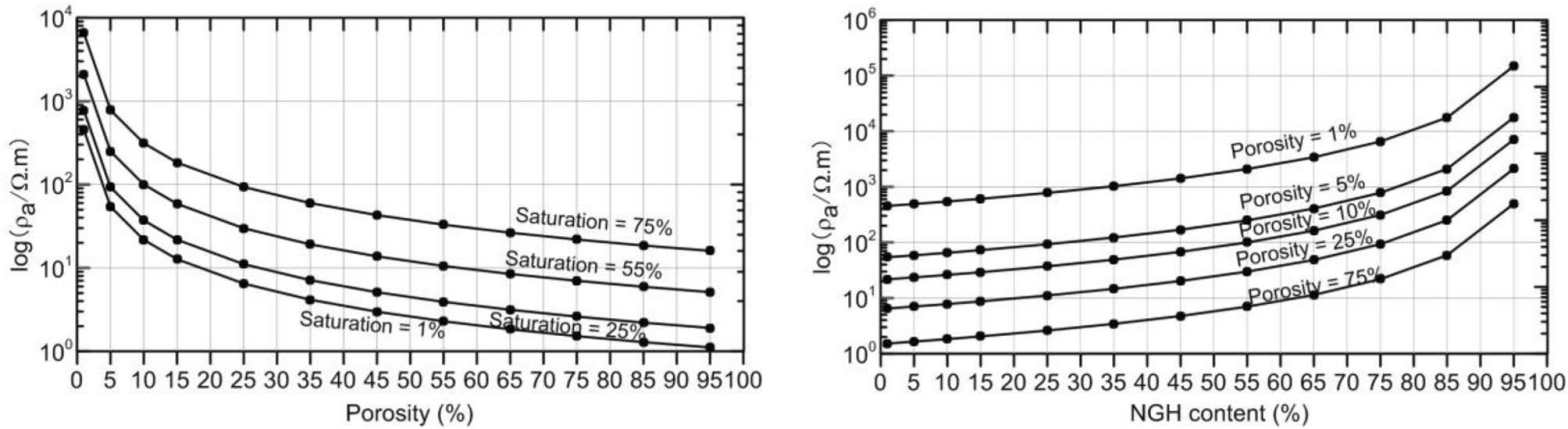
Resistivity as a function of porosity (left) and NGH saturation (right).

To investigate the relations between resistivity, porosity, and NGH content, we assumed that porosity varied from 1 to 95% and at NGH saturation from 1 to 95% based on known drilling information. According to the porosity and saturation of the NGH in the permafrost area of Qilian Mountain, the resistivity of the reservoirs can be estimated with Eq. (3), as shown in Fig. 6. For constant NGH saturation, the resistivity of the NGH reservoir reduced as the porosity increased from 1 to 95%. Similarly, when the porosity was fixed, the resistivity increased as the NGH saturation increased from 1 to 95%. This indicates that the resistivity of the NGH reservoir is closely related to both porosity and NGH saturation.

It can be seen from Table 1 that the resistivity of the NGH reservoir varies from 24.17 to 396.6 Ω m. It can be found in Fig. 6 that the variation range of porosity and saturation corresponding to this resistivity is 5–20% and 50–70%, respectively.

Figure 6 shows that when the resistivity of the gas hydrate reservoir is higher than 396.6 Ω m, the corresponding porosity will be less than 5% and the saturation will be higher than 70%. It indicates that the reservoir is a low porosity, high saturation reservoir. According to the above analysis, when the porosity is less than 5% and the saturation is higher than 70%, the resistivity parameters of the MTIP method cannot identify and define the NGH reservoirs in the permafrost area of the Qilian Mountains.

**Figure 7:**
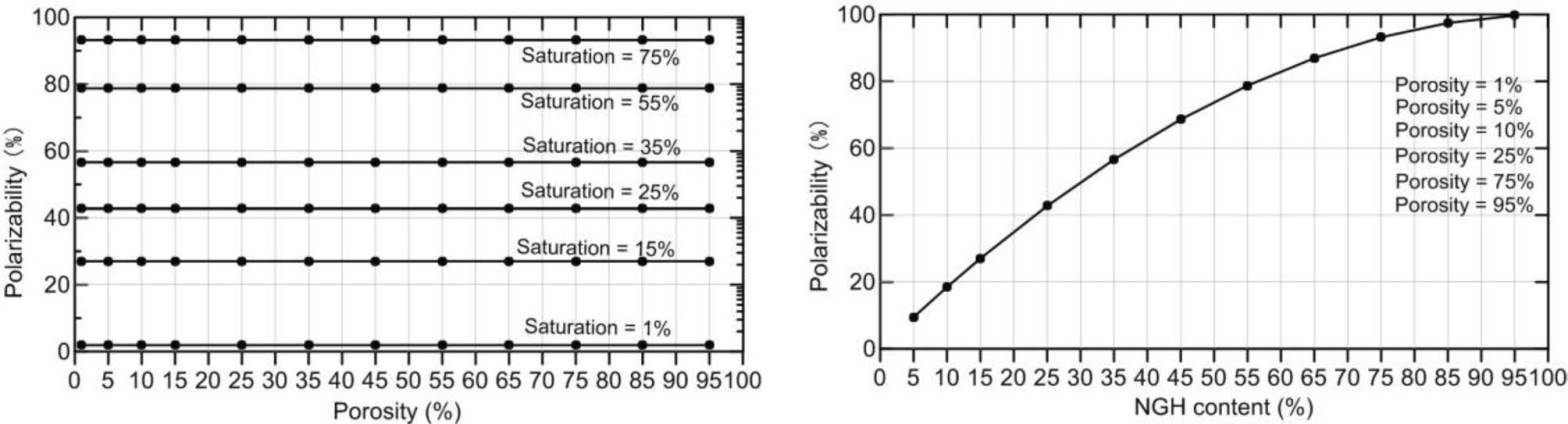
Polarizability as a function of porosity (left) and NGH saturation (right).

The IP can, thus, be calculated, and the polarizability as a function of porosity and as a function of NGH content is shown in Fig. 7. For fixed NGH saturation, the polarizability was constant as the porosity increased from 1 to 95%. However, for a fixed porosity, the polarizability increased as the NGH saturation increased from 1 to 95%. This indicates that the polarizability depends on the NGH content but not on the porosity. The polarizability, thus, indicates the presence of NGH and can guide the subsequent exploration and drilling.

Based on the ranges of the porosity and saturation of the NGH in the permafrost in the Qilian Mountains, porosity and NGH content can be calculated. The storage capacity of NGH can be found by combining the resistivity and polarizability obtained by MTIP inversion. Hence, based on the difference in polarizability between NGH and the surrounding rock, the polarizability of MTIP is suitable for the geophysical exploration of NGH in the Muli area of the Qinhai-Tibetan Plateau.

**Figure 8:**
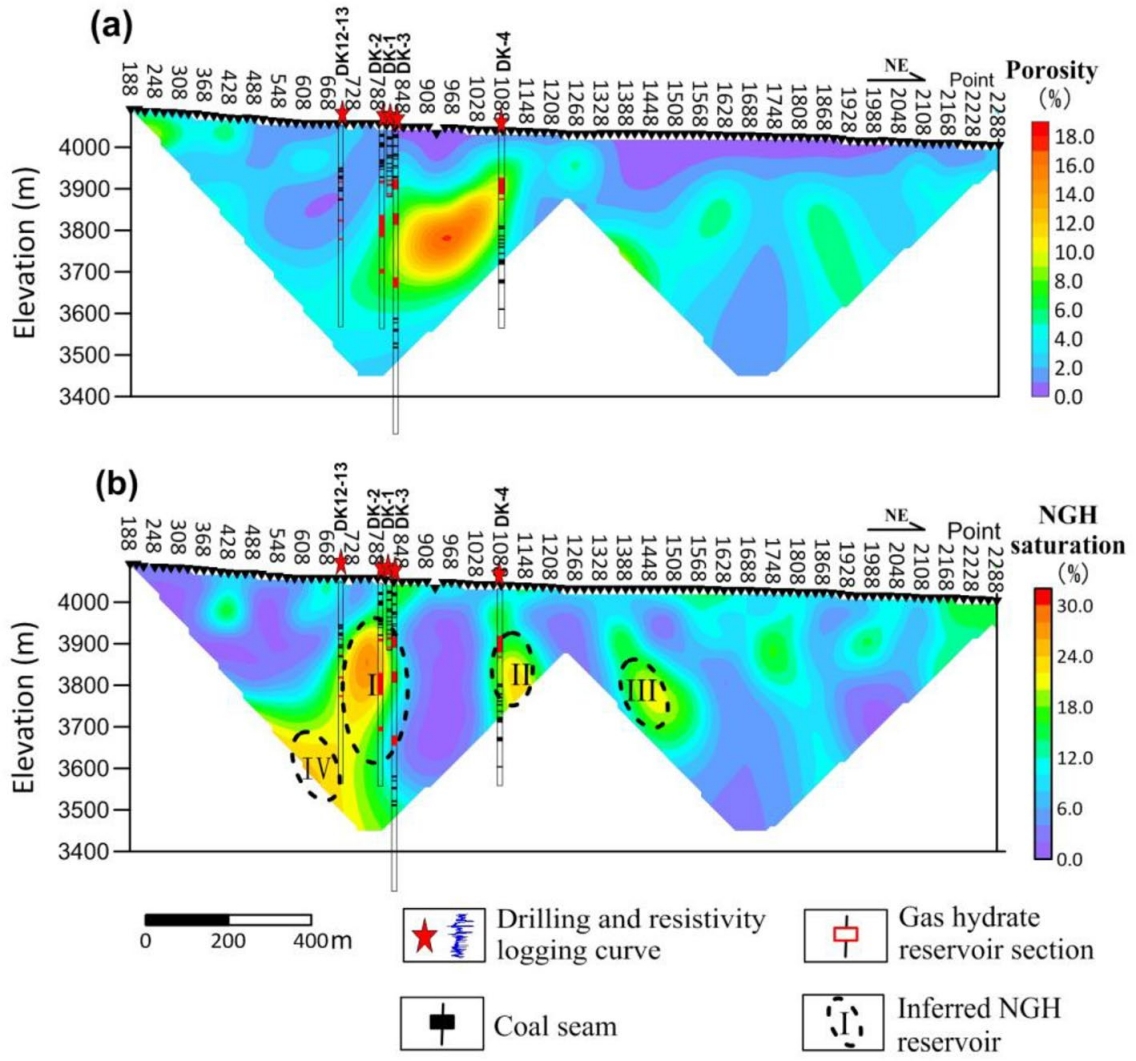
Porosity and NGH saturation sections. (**a**) Section of porosity, (**b**) Section of NGH saturation.

The porosity and NGH saturation can be inverted using the MTIP resistivity and polarizability data using Eqs. (3) and (4). The amplitude of the porosity (Fig. 8a) ranges from 0 to 20%. In the shallow permafrost region, the high resistivity corresponds to low porosity, as low as 1%. Faults at elevations between 3700 and 3900 m have a high porosity, up to 20%. The porosity and resistivity distribution reflect the underground lithological characteristics and fault zones. Similarly, NGH saturation (Fig. 8b) ranges from 0 to 32%. The I, II, and III high saturation anomalies are consistent with the I, II, and III high polarizability anomalies. The IV high saturation anomaly is not in the polarizability section.

According to the resistivity and porosity results, as shown in Figs. 5a and 8a, it can be concluded that the fault zone is characterized by a low-resistivity, high-porosity anomaly. The fault zone is characterized by high polarizability and high NGH saturation, as shown in Figs. 5b and 8b. It can be inferred that the NGH in this region depends on the fault zone. The well-developed fracture is a good channel in which NGH can rise, forming NGH in the low-temperature environment due to the layer of permafrost. The fracture can be inferred from the resistivity. When combined with porosity, the degree of fracture development can be determined. The polarization and saturation indicate the presence of NGH.

## Conclusions

The electrical and lithological characteristics of gas hydrate reservoirs were studied for use in exploring the presence of NGH in the Muli area, and the presence of NGH in the fault zone was evaluated using the NGH saturation based on MTIP data inversion. The main conclusions are as follows:

1. The porosity of a rock controls its resistivity, and NGH saturation and polarizability are in nice agreement. Three polarizability and saturation anomalies have been recognized as known NGHs, and one saturation anomaly has been identified as a potential NGH. The inferred permafrost overburden thickness and the five south-dipping faults provide a favourable geological environment for hydrate movement and storage.
2. Based on the analysis of the physical properties of underground NGH reservoirs. The resistivity of the sandstone reservoir containing hydrate is 2–3.5 times that of the surrounding rock, and its thickness is thin, so it is difficult to identify the hydrate by resistivity alone, but obtaining resistivity parameters from MTIP can delineate the thickness of the permafrost layer and the fracture distribution to infer the underground NGH source and transport channel.
3. A summary of electrical and lithological characteristics can be used to evaluate the existence of the NGH, The MTIP measurement results are basically consistent with the borehole logging data, and the polarizability and saturation can assess the possibility of the existence of the NGH, which provides an important basis for the identification and distribution of natural gas hydrate reservoirs.

## Data Availability

Data associated with this research is available and can be obtained by contacting the corresponding author.
